# Supplementary material for: Spatiotemporal trends and coordination of agricultural carbon efficiency in the Yangtze River Economic Belt and Yellow River Basin, China: An analysis of influencing factors and green finance integration
Source: PLoS One. 2024 Aug 29;19(8):e0308399. doi: 10.1371/journal.pone.0308399 (PMC11361668; doi:10.1371/journal.pone.0308399)
Supplement: S1 File — (DOCX) [file pone.0308399.s001.docx]

S1 data1 (XLSX)

<https://doi.org/10.6084/m9.figshare.25918564>

S2 data2 (XLSX)

<https://doi.org/10.6084/m9.figshare.25918582>

S3 data3 (XLSX)

<https://doi.org/10.6084/m9.figshare.25918618>
