# Supplementary material for: Spatiotemporal trends and coordination of agricultural carbon efficiency in the Yangtze River Economic Belt and Yellow River Basin, China: An analysis of influencing factors and green finance integration
Source: PLoS One. 2024 Aug 29;19(8):e0308399. doi: 10.1371/journal.pone.0308399 (PMC11361668; doi:10.1371/journal.pone.0308399)
Supplement: Attachment — Submitted filename: Comments.docx [file pone.0308399.s002.docx]

1. Please add line numbers in the manuscript. The current version does not have line numbers and cannot clearly describe the specific areas that need to be modified in the article.
2. Is there no citation of references in the introduction section? Please add relevant literature references.
3. “used the IPCC carbon emission coefficient method to measure ACE in Shandong Province from 2000 to 2020”, the u in “used” needs to be capitalized.
4. It is strongly recommended to organize the abbreviations of nouns in the text into a table at the beginning of the manuscript.
5. “with Heze City ranking first..”. An additional period has been added.
6. The literature review only briefly lists the research and does not provide its own summary and viewpoint, which is not a qualified literature review. Please revise and summarize carefully
7. Before 2015, agricultural production, especially planting activities, in the Yangtze and Yellow River basins were still mainly focused on small-scale farming, while intensive agricultural production was mainly concentrated in the northeast and northwest China. However, the majority of young and middle-aged people in rural areas enter cities in search of higher incomes and rarely directly participate in agricultural activities. So why choose indicator 3 for human resources and indicator 4 for green production efficiency in enterprises?
8. The results and discussion suggestions are separated, and the current fifth chapter is too long.
9. Please carefully check the format and content expression of the article. Currently, some expressions are too colloquial and there are issues with inconsistent formatting.
10. Please modify the image color scheme and table format, as the image title is too short.
