# Supplementary material for: Spatiotemporal trends and coordination of agricultural carbon efficiency in the Yangtze River Economic Belt and Yellow River Basin, China: An analysis of influencing factors and green finance integration
Source: PLoS One. 2024 Aug 29;19(8):e0308399. doi: 10.1371/journal.pone.0308399 (PMC11361668; doi:10.1371/journal.pone.0308399)
Supplement: Attachment — Submitted filename: Response to Reviewers.docx [file pone.0308399.s003.docx]

Dear editor and reviewers,

Thank you for your review. We have revised the manuscript in accordance with the comments and marked all the amends on our revised manuscript. The following is a list of responses to each suggestion. (reviewers’ comments in black, our replies in blue)

Reviewer #2: The author did not find any replies to his comments in the attached manuscript as the authors stated. The reviewer insists on the writing of each of his comment attached with the response of author.

Response: Dear Reviewer 2, in the first revision version, according to the requirements of Plos One, we have submitted three revised documents: (1) A revised letter that responds to each point raised by the academic editor and reviewer (s) labeled 'Cover letter. (2) A marked-up copy of the manuscript highlighting changes made to the original version, labeled 'Revised Manuscript with Track Changes'. (3) An unmarked version of the revised paper without tracked changes, labeled 'Manuscript'.

Previously, we placed Response to Reviewers in the "cover letter" folder. This time, we have placed Response to Reviewers in a dedicated "Response to Reviewers" folder. It is at the end of the system generated file "PONE-D-24-07132-R2".

In addition, in order to have a clearer understanding of the modifications for you, we have acceptance of amendments made to Reviewer 1 (another reviewer) in the Revised Manuscript with Track Changes document. Therefore, this version of the Revised Manuscript with Track Changes is only based on your previous comment.

We also have attached the response to your comment point-to-point from last time below.

We apologize for any inconvenience caused and thank you again for your review. Thank you!

Reviewer #2: The study have valuable insights into the spatiotemporal dynamics, influencing factors, and coupling coordination of agricultural carbon emission efficiency in the YEB and YRB regions in China. It highlights the importance of technological progress, human capital quality, economic development level, government regulation, and scientific and technological innovation ability in influencing the coordination between ACE efficiency and green finance. These findings have implications, aiming to promote sustainable agricultural systems and achieve carbon neutrality in China's river basins.

However, there are some comments and inquiries as follows:

Major comments

-------------------------

1-What is the novelty in your article?

Response: Dear Reviewer 2, we appreciate your careful review and constructive suggestions regarding our manuscript. In fact, in section 2.5 of the article, we have elaborated on the novelty of the article. However, we may not have a clear expression of the novelty of the article, so we have modified it to make the expression clearer. The specific modification content is as follows:

Different from the existing research literature, the contributions of this paper are as follows: (1) Since the Yangtze River and Yellow River basins are extensive in scope and have certain geographical environmental characteristics, it is better to take the basin as the research object than the regional study of administrative division, so as to better study the carbon emission under the difference of physical geographical environment, analyze the difference between the north and the south from the perspective of the basin, and provide targeted suggestions for the development of different regions.

(2) Existing literature studies focus on the coupling and coordination analysis of carbon emission efficiency, economic development and industrial structure optimization. This paper innovatively analyzes the coupling and coordination analysis of carbon emission efficiency and green finance.

2-How is your article different from the following articles:

a-Coupling Coordination and Spatiotemporal Dynamic Evolution between Agricultural Carbon Emissions and Agricultural Modernization in China 2010–2020

b-The Coupling and Coordination of Agricultural Carbon Emissions Efficiency and Economic Growth in the Yellow River Basin, China

c-Conflict or Coordination? Analysis of Spatio-Temporal Coupling Relationship between Urbanization and Eco-Efficiency: A Case Study of Urban Agglomerations in the Yellow River Basin, China

Response: Thank you for your detailed comments. **Paper a** studies the coupling coordination degree between agricultural carbon emissions and agricultural modernization in China from 2010-2020 from the perspective of administrative region. **Paper b** studies the coupling coordination degree between agricultural carbon emission efficiency and economic growth in 30 cities in the Yellow River Basin from 2010 to 2020, and the Dagum Gini coefficient and kernel density estimation methods were adopted in order to comprehensively examine the spatial differences, as well as the dynamic evolution pattern of the coupled coordination in the YRB. **Paper c** studies the spatial correlation analysis and coupling coordination analysis of urbanization efficiency and ecological efficiency of 7 urban agglomerations in the Yellow River Basin from 2006 to 2019.

**In our paper**, the research object is the Yangtze River Economic Belt and the Yellow River basin, and the research content is the coupling and coordination analysis of agricultural carbon emission efficiency and green finance in the two basins and its influencing factors, which is quite different from the above three partial papers.

The specific differences are shown in the table below:

|  | Paper a | Paper b | Paper c | Our paper |
| --- | --- | --- | --- | --- |
| Research object | Provinces of China | 30 cities in the YRB | seven urban agglomerations in the YRB | 19 regions in the YEB and YRB |
| Research content | CCD between agricultural carbon emissions and agricultural modernization | CCD between agricultural carbon emission efficiency and economic growth | spatial correlation analysis and CCD of urbanization efficiency and ecological efficiency | CCD between agricultural carbon emission efficiency and green finance and its influencing factors |

3-The title needs to be upgraded. What about this title “Spatiotemporal Trends and Coordination of Agricultural Carbon Efficiency in YEB and YRB River Basins, China: An Analysis of Influencing Factors and Green Finance Integration”

What about the investigations after 2020?

Response: Thank you for your detailed comments. Thank you for the paper title. It is more consistent with the content of the paper, and we have applied the paper title in the original paper.

The data in this paper was collected in 2023. Due to the lag of the data, although some data were updated to 2021, there were still some data that were not updated. Moreover, the data from 2001 to 2020 were analyzed to facilitate comparative analysis by time period.

4-What about the depth of coupling coordination?

I mean the study evaluates CCD between ACE efficiency and green finance but not investigates deeply the specific mechanisms, policies, or strategies that can enhance coordination or improve agricultural carbon emission efficiency.

Response: Thank you for your detail comments. In Section 5.5 of the paper, the specific mechanisms for improving coupling coordination are:

When the CCD is at the disorder grade, improving the quality of human capital, the level of economic development, the ability of scientific and technological innovation, and stimulating the macro-control of the government can effectively improve the CCD of the two systems. However, when the CCD of the two systems enters the coordination grade, improving the level of economic development, scientific and technological innovation ability, and stimulating the macro-control of the government cannot effectively improve the CCD, but only improving the quality of human capital can effectively improve the CCD of the two systems. Therefore, the most effective way to improve the coupling coordination degree between agricultural carbon emission efficiency and green finance is to improve the quality of human capital.

In Chapter 7 of the paper, the specific strategies for improving coupling coordination are:

On the one hand, more talents can be attracted to stay in rural areas, and the quality of human capital in rural areas can be improved through policy inclination and assistance policies. The application of advancing research results, policies, and concepts by agricultural talents can not only promote the development of green finance by enabling more environmentally friendly projects to be developed through the financing of green finance but also improve the efficiency and quality of agricultural production, improve the efficiency of carbon emission, and promote the coupling and coordinated development of the two systems. On the other hand, training high-quality farmers, a precise selection of cultivation objects, strengthening the construction of the cultivation system and promoting the quality and efficiency of the cultivation work (in the original paper).

Minor comments

_________________

In Figure 2, the X-axis title is in Chinese language.

In Table 3, Capitalize the items in “Carbon emission resources”.

In Table 6, add the title of year column.

Response: Thank you for your detail comments. We are sorry that there are some errors in the chart details. We have corrected the error you pointed out in the original text. In Figure 2, we convert the X-axis into English. In Table 2-4, We changed “Carbon emission sources” into “Carbon Emission Resources”. In Table 7(Since Table 1 is new, it becomes Table 7), we added the title of year column. Please refer to the revised paper for details.
